# Composites of Quasi-Colloidal Layered Double Hydroxide Nanoparticles and Agarose Hydrogels for Chromate Removal

**DOI:** 10.3390/nano6020025

**Published:** 2016-01-26

**Authors:** Gyeong-Hyeon Gwak, Min-Kyu Kim, Jae-Min Oh

**Affiliations:** Department of Chemistry and Medical Chemistry, College of Science and Technology, Yonsei University, Wonju, Gangwondo 26493, Korea; henny@yonsei.ac.kr (G.-H.G.); ipz9rv@yonsei.ac.kr (M.-K.K.)

**Keywords:** quasi-colloidal nanoparticle, electrophoretic method, layered double hydroxide, agarose, hydrogel, chromate removal, Freundlich adsorption

## Abstract

Composite hydrogels were prepared that consisted of quasi-colloidal layered double hydroxide (LDH) nanoparticles and agarose via the electrophoretic method, starting from three different agarose concentrations of 0.5, 1, and 2 wt/v%. The composite hydrogel was identified to have a uniform distribution of LDH nanoparticles in agarose matrix. Microscopic studies revealed that the composite hydrogel had a homogeneous quasi-colloidal state of LDHs, while the simple mixture of LDH powder and agarose hydrogels did not. It was determined that agarose concentration of the starting hydrogel did not significantly influence the amount of LDH that developed in the composite. The chromate scavenging efficiency of the composite hydrogel and corresponding agarose or mixture hydrogel was evaluated with respect to time, and chromate concentration. In general, the composite hydrogels exhibited much higher chromate removal efficacy compared with agarose or mixture hydrogels. Through estimating chromate adsorption by LDH moiety in the composite or mixture hydrogel, it was suggested that the agarose component facilitated the stability and dispersibility of the quasi-colloidal state of LDH nanoparticles in the composite resulting in high adsorption efficacy. From Freundlich isotherm adsorption fitting, composites were determined to possess beneficial cooperative adsorption behavior with a high adsorption coefficient.

## 1. Introduction

Layered double hydroxides (LDHs) are a family of two-dimensional layered structures and are known to have a high anionic removal property due to its strong positive surface charge and exchangeable interlayer anions [1]. The general chemical formula of LDHs is denoted as M(II)_1−*x*_M(III)*_x_*(OH)_2_(A*^n^*^−^)*_x_*_/*n*_·*m*H_2_O (M(II): divalent metal cation, M(III): trivalent metal cation, A*^n^*^−^: anionic species with *n*^−^ charge, 0 < *x* < 1, *m*: interlayer water quantity) and can be divided into two parts: the positive layers, [M(II)_1−*x*_M(III)*_x_*(OH)_2_]*^x^*^+^ and the interlayer components, [(A*^n^*^−^)*_x_*_/*n*_·*m*H_2_O] [2]. As opposed to other layered materials like smectite clays (cationic exchange capacity ~100 meq/100 g) [3], LDHs are reported to have a high anionic exchange capacity of ~450 meq/100 g [4]. Thus, extensive studies have been carried out to develop an LDH-based anionic scavenging system. For example, Ulibarri *et al.* studied the adsorption of 2, 4, 6-trinitrophenol and dodecylbenzylsulfonate on MgAl-LDH, depending on the interlayer anions and crystallinity of LDH [5]. Materials capable of removing U(VI) oxocarbonate from aqueous media were developed utilizing ZnAl-LDH and several chelating moieties, exhibiting a maximum removal efficacy of 100 μmol U(VI)/g LDH [6].

Although LDHs themselves possess several advantages in anion adsorption, there still remain drawbacks for practical application. LDH nanoparticles in powder state often form agglomerates due to strong inter-particle interactions [7,8], which not only hinders their dispersibility in aqueous media but also reduces adsorption sites on LDHs’ surface. Even though LDH nanoparticles are perfectly dispersed in liquid media and adsorb anionic pollutants, they can give rise to secondary pollution if they cannot be collected. In order to solve the collection problem, several strategies have been suggested, such as introduction of magnetic properties to the LDHs [9,10,11] and fabrication of monoliths containing LDH moieties [12,13]. Composites prepared by the self-assembly of superparamagnetic Fe_3_O_4_ nanoparticles and LDH are reported, which showed a high adsorption property for Congo red dye and were easily separated by a magnet [9]. Hibino prepared several kinds of monolith-type agarose-LDH composites as anion scavengers [13,14,15]. In these instances, delaminated LDHs suspended in water were mixed with a hot agarose solution and cooled down to obtain a monolith. This composite system was reported to show high removal efficiency for several anions such as SO_4_^2−^, I^−^ and HPO_4_^2−^, while maintaining the possibility of easy collection after water treatment. Another example of a monolith containing LDH is reported by Tokudome *et al.* They prepared an LDH-based monolith having interconnected hierarchical channels through a sol-gel process utilizing poly (ethylene oxide) and polypropylene oxide [12].

In this regard, we endeavored to develop a monolith containing quasi-colloidal LDH nanoparticles, which were homogenously dispersed in an agarose based hydrogel. According to our previous study, the electrophoretic preparation [16] was effective in obtaining a homogeneous distribution of metal hydroxide nanoparticles inside the hydrogel [17]. In this preparative method, the hydrogel monolith was first prepared and then an electrical force transported metal cations or anionic species into the gel to uniformly generate metal hydroxide nanoparticles throughout the matrix. In this study, to obtain LDH-agarose composites by the electrophoretic method, we first prepared hydrogels having an agarose concentration of 0.5, 1 and 2 wt/v%, respectively. Both cationic (Mg^2+^, Al^3+^) and anionic (OH^−^, CO_3_^2−^) precursors for LDH were electrically transferred into each hydrogel to yield Mg_2_Al(OH)_6_(CO_3_^2−^)_0.5_-LDH nanoparticles in between agarose chains. In the composite, nanoparticles would be dispersed in aqueous media, while their mobility was limited as a result of being entrapped in the hydrogel matrix. Therefore, the LDH nanoparticles in the composite could exist in a quasi-colloidal state. We verified the homogeneous generation of LDH nanoparticles in the composites via electron microscopy; while, agarose-LDH mixtures exhibited an inhomogeneous distribution and resulted in agglomeration of LDHs. Chromate (CrO_4_^2−^) removal behavior of the composites were evaluated with respect to time, and chromate concentration. The adsorption isotherms were analyzed using the Freundlich adsorption model [18]. Furthermore, we investigated the effects of quasi-colloidal state of LDH nanoparticles and its homogeneity on the chromate removal efficacy of the composites. We also examined the effect of agarose concentration in the starting hydrogel on the amount of electrophoretically-generated LDH, quasi-colloidal state of LDH nanoparticles, and chromate scavenging ability of the composites.

## 2. Results and Discussion

### 2.1. Characterization of Electrophoretically Prepared LDH-Agarose Composites

**Figure 1:**
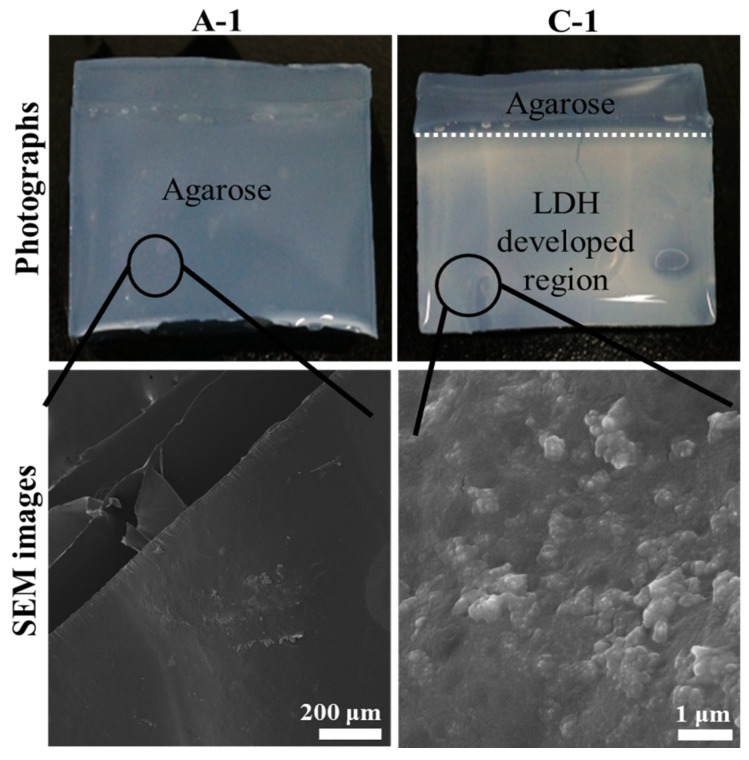
Photographs and scanning electron microscopic (SEM) images of agarose only hydrogel (1 wt/v% agarose concentration, **A-1**) and Layered double hydroxide (LDH)-agarose composite hydrogel (1 wt/v% agarose concentration, **C-1**). For SEM images, both A-1 and C-1 were dehydrated to film.

Figure 1 exhibited photographs and scanning electron microscopic (SEM) images of electrophoretically-prepared LDH-agarose (1 wt/v% agarose concentration) composite, namely C-1, along with those images of agarose (1 wt/v%) hydrogel (A-1). Photographs were taken with water containing the hydrogel state and SEM images were obtained with dehydrated hydrogel films. As shown in the photograph, C-1 contained a translucent white region below the white dotted line, while A-1 was homogeneously transparent. As we filled the anionic and cationic precursor solution to the white dotted line during electrophoresis, LDH nanoparticles might develop in that region. As the white color in the LDH developed region was fairly homogeneous, the formation of LDH nanoparticles was considered to occur uniformly throughout the hydrogel, as reported previously with hydrozincite [17]. Thus, we could conclude that LDH nanoparticles existed in a colloid-like state being entrapped by the agarose moiety. According to this result, we could suggest the formation mechanism of LDH nanoparticles. Metal cations (Mg^2+^ and Al^3+^) in anodic compartment and anionic species (CO_3_^2−^ and OH^−^) in cathodic compartment moved to opposite directions, cathode and anode direction, respectively, upon potential application. It is thought that those ionic species (cations and anions) met inside the agarose hydrogel, resulting in development of metal hydroxycarbonate precipitates. As a result, LDH nanoparticles were formed inside the hydrogel.

The SEM image of C-1 (Figure 1) showed small particles that were on the magnitude of ten nanometers in size and were homogeneously distributed through the smooth surface (agarose polymer matrix). On the other hand, the agarose only (A-1) showed a flat and clean surface, which was attributed to the strong intermolecular interaction of amorphous organic moiety [19], and no significant particles were found. Compared with LDH nanoparticles in the powder form (Figure S1), those in C-1 showed a blurred grain boundary and blunt edges, similar to that found in inorganic nanoparticles coated with organic moiety [20]. Therefore, it could be suggested that every LDH nanoparticle was homogeneously coated with polysaccharide chains of agarose. This could be possible by two means. First, the LDH had a positive surface charge, which could strongly interact with the partial negative charges of the polysaccharides [21]. Second, the crystal growth of the LDH nanoparticle occurred homogeneously among the agarose matrix and, thus, the particles readily interacted with adjacent polysaccharide.

**Figure 2:**
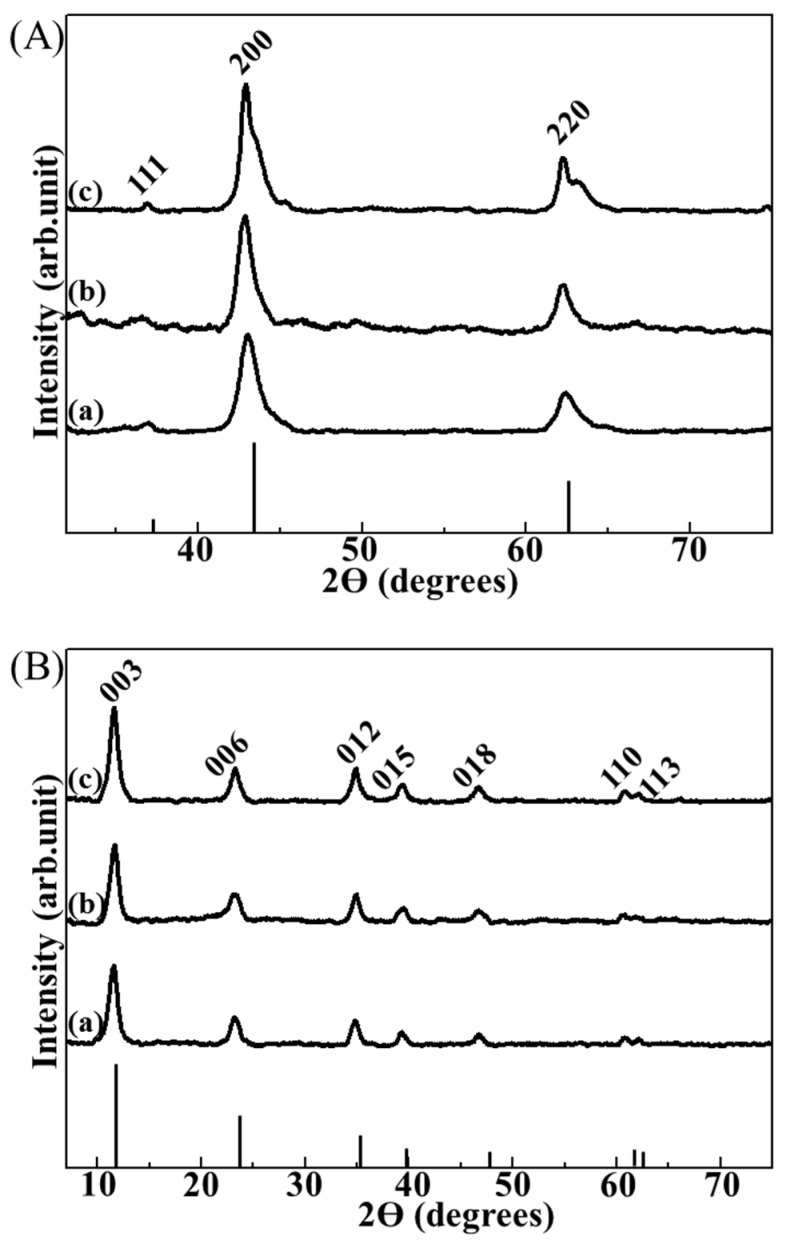
X-ray diffraction (XRD) patterns of (**A**) calcined composites and (**B**) reconstructed ones for (**a**) LDH-agarose composite hydrogel (0.5 wt/v% agarose concentration, C-0.5), (**b**) LDH-agarose composite hydrogel (1 wt/v% agarose concentration, C-1) and (**c**) LDH-agarose composite hydrogel (2 wt/v% agarose concentration, C-2), respectively. XRD patterns of the calcined composite (**A**) showed a typical phase of periclase (JCPDS No. 71-1176), and those of reconstructed ones (**B**) exhibited the layered double hydroxide (LDH) phase (hydrotalcite, JCPDS No. 14-0191). The vertical lines under the XRD patterns are the corresponding JCPDS patterns.

Powder X-ray diffraction (XRD) analyses were employed in order to indirectly confirm that the small particles found in the composite were LDHs, utilizing reconstruction property of LDHs [22]. LDHs (Mg_2_Al(OH)_6_(CO_3_^2−^)_0.5_) are well known to transform into a mixed metal oxide (MMO) through dehydroxylation and decarbonation when they are calcined at ~600 °C. Then MMOs could recover their original LDH structure by treatment with water and carbonate ions, which is referred to as reconstruction [23]. In this study, three kinds of LDH-agarose composites were electrophoretically-prepared starting with 0.5, 1 and 2 wt/v% agarose hydrogel and those composites were named C-0.5, C-1, and C-2, respectively, where the number represents concentration of agarose in the hydrogel. All of the XRD patterns of the calcined composites showed the periclase phase (JCPDS No. 71-1176) [24], which is the typical form of calcined Mg_2_Al(OH)_6_(CO_3_^2−^)_0.5_-LDH (Figure 2A). The XRD patterns of the calcined composites after treatment with water and carbonate ions (Figure 2B) exhibited well-crystallized hydrotalcite (Mg_2_Al(OH)_6_(CO_3_^2−^)_0.5_-LDH) phase (JCPDS No. 14-0191) [25]. These results strongly suggested that the particles found in each composite were LDH nanoparticles.

**Figure 3:**
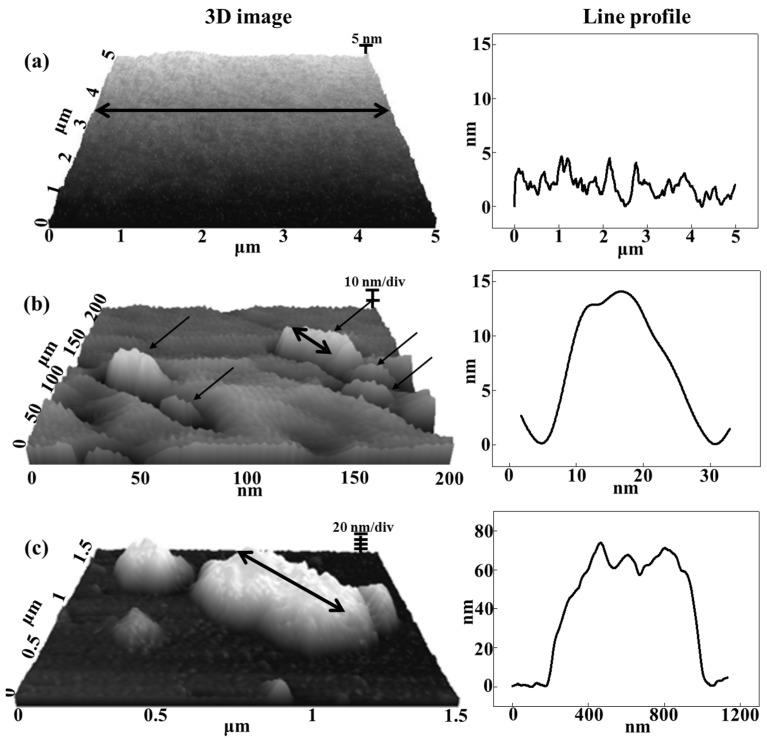
Three-dimensional atomic force microscopic (AFM) images and corresponding line profiles along the double-headed arrow for (**a**) agarose only hydrogel (1 wt/v% agarose concentration, A-1), (**b**) LDH-agarose composite hydrogel (1 wt/v% agarose concentration, C-1) and (**c**) LDH-agarose mixture hydrogel (1 wt/v% agarose concentration, M-1), respectively. Single-headed arrows in (**b**) stand for singe LDH nanoparticles distributed in agarose matrix.

Given that the SEM measurement was carried out with a dehydrated composite film, LDH particles could gather together during dehydration, resulting in LDH-agarose agglomerates (Figure 1). To verify that the inter-particle distance of the LDH was sufficient to form a colloidal state in the composite, we carried out atomic force microscopy (AFM) measurements on the composite (C-1) containing a water moiety (Figure 3). For comparison, we also measured the surface topology of the agarose hydrogel (A-1) and LDH-agarose mixture (M-1). Here, the LDH-agarose mixture stands for the hydrogel monolith prepared by simply mixing a hot agarose solution and powdered LDH. The amount of agarose and LDH was set to be equivalent to that of the corresponding composite. The number at the end of the sample name, like in the case of the composites, represents the concentration of agarose in hydrogel. The AFM image of A-1 was fairly flat and smooth. The composite (C-1) showed several particles (arrows in Figure 3b) sparsely distributed on a flat surface. The line profile of the single LDH nanoparticle showed a particle size approximately 20 nm (Figure 3b), which corresponded to the particle size found in SEM (Figure 1). In contrast, M-1 showed relatively large lumps of approximately micron-sized particles, suggesting that the powdered nanoparticles was not homogeneously distributed in the agarose hydrogel matrix through simple mixing. Thus, we proposed that the electrophoretically-prepared composite contained LDH nanoparticle moieties in a quasi-colloidal state. In the colloid, each particle was stably suspended in media through strong inter-particle repulsion [26]. Similarly, the LDH nanoparticles in the composite stayed in the agarose hydrogel matrix maintaining an appropriate inter-particle distance.

### 2.2. Quantification of LDH in Composites

To investigate whether the agarose concentration in the hydrogel influenced the amount of generated LDH, we measured the mass of the calcined composite, where only MMO exist. As summarized in the Table 1, the mass of the calcined composite was 0.0360, 0.0316, and 0.0322 g for C-0.5, C-1, and C-2, respectively. As we used an Mg/Al ratio of 2/1 in the metal precursor solution, LDHs with formula Mg_2_Al(OH)_6_(CO_3_)^2−^_0.5_ (formula weight = 207.64 g/mol) were expected to be formed in the composites. Upon calcination, the LDH would be converted to MMO with the formula Mg_2_AlO_3.5_ (formula weight = 131.59 g/mol). Applying the formula weight ratio to the mass of the MMO, we could estimate the mass of the LDH in the composites as 0.0568, 0.0499, and 0.0508 g for C-0.5, C-1, and C-2, respectively, showing no tendency towards the LDH amount depending on the starting agarose concentration.

**Table 1:** Mass of each calcined composite and estimated mass of layered double hydroxide (LDH) in composite. Mass of the LDH was calculated taking into account that the Mg_2_Al(OH)_6_(CO_3_^2−^)_0.5_-LDH transformed into Mg_2_AlO_3.5_ and that the agarose moiety was decomposed upon calcination.

Samples	C-0.5	C-1	C-2
Mass of calcined composite (g)	0.0360	0.0316	0.0322
Estimated mass of LDH in composite (g)	0.0568	0.0499	0.0508

### 2.3. Evaluation of Chromate Removal Efficacy of Composite and Effect of Quasi-Colloidal LDH

The anion scavenging ability of the composite, which was thought to contain quasi-colloidal LDH nanoparticles, was evaluated by time and concentration-dependent chromate (CrO_4_^2−^) removal experiments. In order to determine the role of quasi-colloidal LDH nanoparticles in the composite on anion removal, the same experiments were also carried out on agarose only hydrogels and LDH-agarose mixture hydrogels.

**Figure 4:**
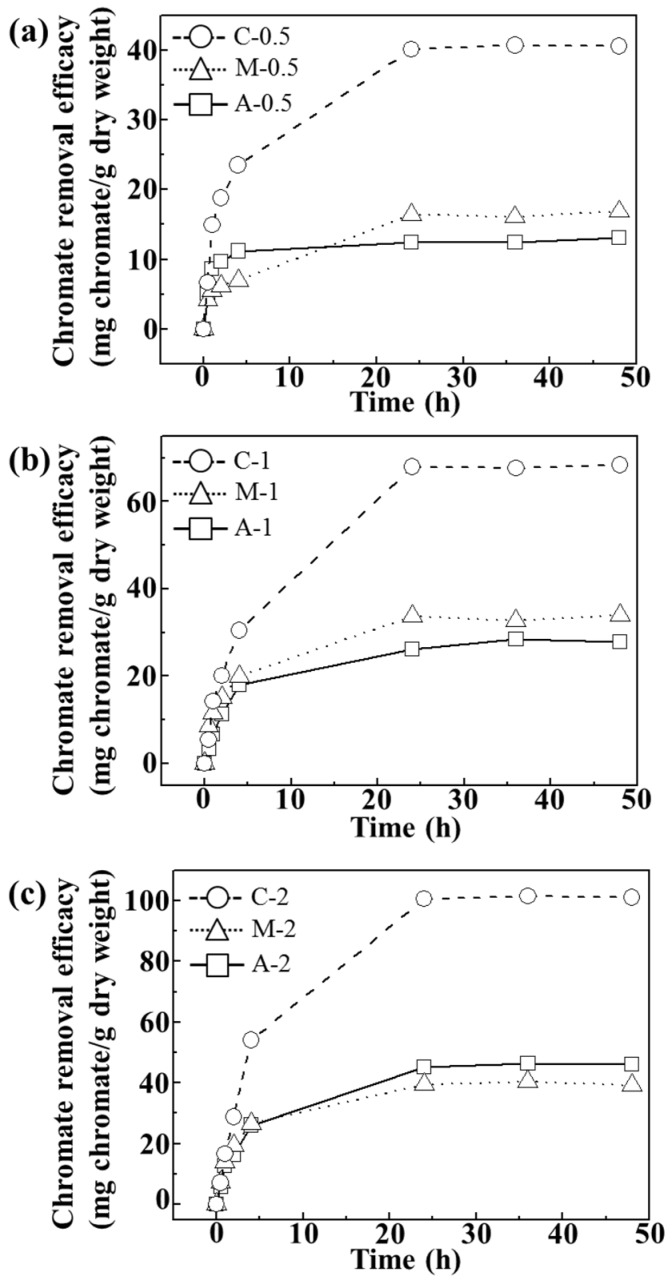
Time-dependent chromate removal efficacy (mg chromate/g dry weight) of agarose only (A), LDH-agarose mixture (M) and LDH-agarose composite (C) samples for (**a**) 0.5 wt/v%; (**b**) 1 wt/v%; and (**c**) 2 wt/v% agarose-based materials. The experiments were carried out at initial chromate concentration of 400 ppm and at pH ~7.5.

As shown in Figure 4, we verified that the adsorption reached equilibrium after 24 h at pH ~7.5. Figure 4a–c showed that the chromate removal efficacy increased as the concentration of agarose hydrogel increased. Composites showed the highest removal efficacy (~41, ~68, and 101 mg CrO_4_^2−^/g dry weight) suggesting that homogeneous distribution of LDH nanoparticles in the composites facilitated chromate removal.

**Figure 5:**
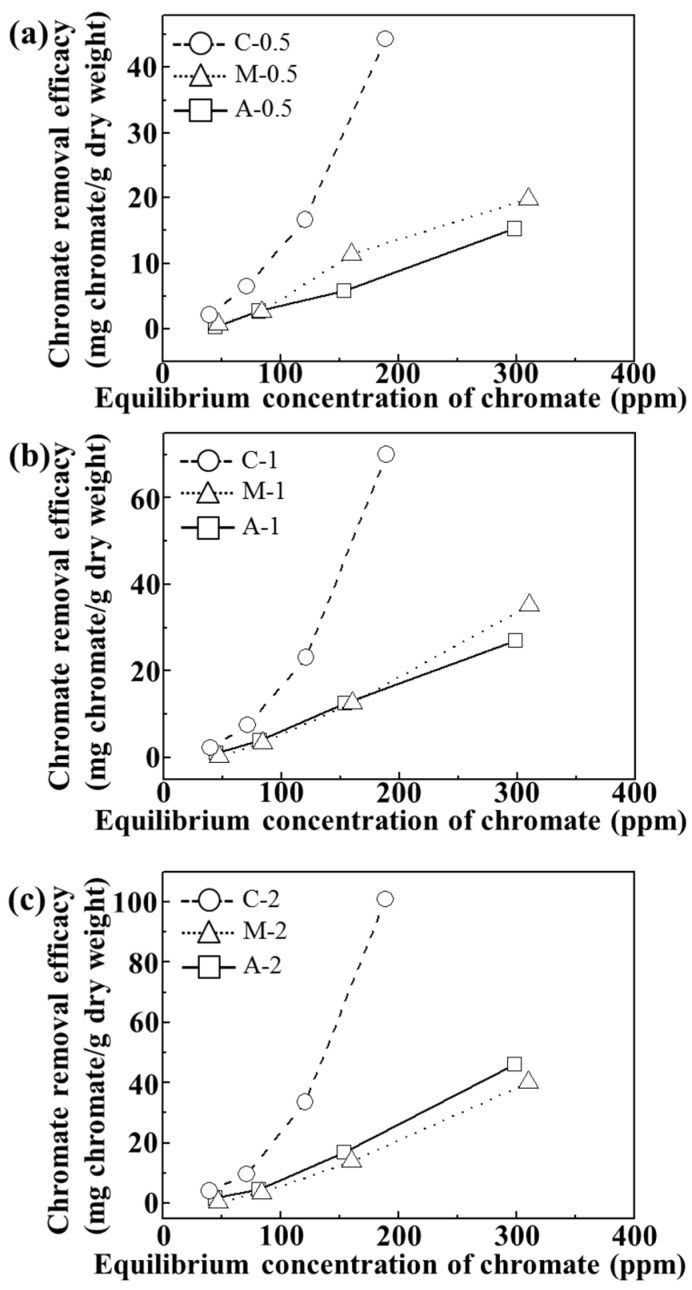
Isotherms of chromate removal efficacy (mg chromate/g dry weight) of agarose only (A), LDH-agarose mixture (M) and LDH-agarose composite (C) samples for (**a**) 0.5 wt/v%; (**b**) 1 wt/v%; and (**c**) 2 wt/v% agarose-based materials. The experiments were carried out at pH 7.2 ± 0.3 after 24 h.

For adsorption isotherm examination, we utilized 24 h of incubation time and pH value of ~7. As shown in Figure 5, all the tested samples showed concentration dependent chromate removal efficacy. The maximum removal efficacy at 310, 250 and 188 ppm equilibrium chromate solution was ~45, ~70, and 100 mg CrO_4_^2−^/g dry weight for C-0.5, C-1, and C-2, respectively. On the other hand, both A and M exhibited maximum removal of ~20, ~35, and 46 mg CrO_4_^2−^/g dry weight for 0.5, 1, and 2 wt/v% hydrogel based samples, respectively. Although the M sample contained an equivalent amount of LDHs (which have anion scavenging capacity), compared with C samples, they did not show any significant advantage of LDHs. In M samples, the LDH particles were agglomerated into a micron-sized particle (Figure 3) and, thus, their surface could not fully play the role of an anion adsorption site. In contrast, the composites, where LDH nanoparticles were homogenously distributed in a quasi-colloidal state, could take maximum advantage of the LDH surface as the anion adsorption site.

**Figure 6:**
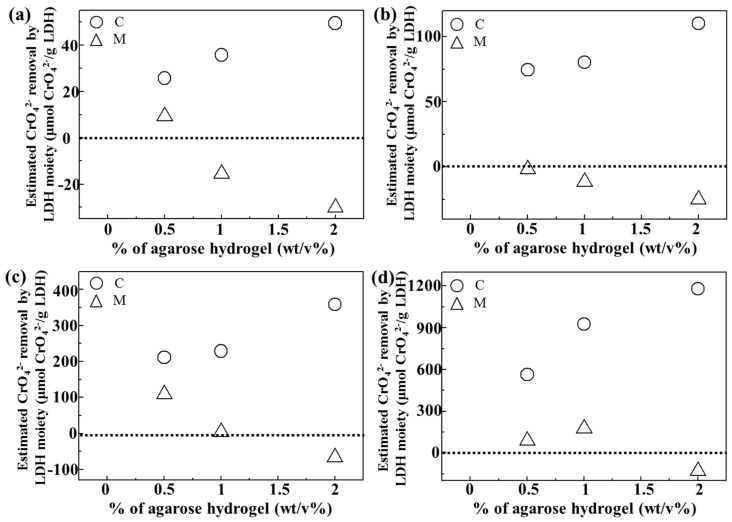
Estimated chromate removal efficacy (μmol/g) by LDH moiety in LDH-agarose composite (C) and LDH-agarose mixture (M) samples at initial chromate concentration of (**a**) 50 ppm, (**b**) 100 ppm, (**c**) 200 ppm, and (**d**) 400 ppm, respectively. For calculation, chromate removal by C or M sample was subtracted by the chromate removal by agarose only at the same chromate concentration.

In order to quantitatively evaluate the role of LDH in either C or M samples, we computed chromate removal by the LDH moiety itself by subtracting the chromate removal resulting from agarose (Figure 6). As shown in Figure 5, the agarose hydrogel itself seemed to have a chromate removal ability, possibly through concentration-gradient diffusion. Thus, the removal of chromates by agarose only (A-0.5, A-1, and A-2) was subtracted from that of the composites (C-0.5, C-1, and C-2) and the mixtures (M-0.5, M-1 and M-2), respectively. The baseline at 0 in Figure 6 meant no adsorption effect by LDH itself. As expected, all composites exhibited positive values of chromate removal by the LDH moiety at all the tested chromate concentration. Furthermore, the anion scavenging functionality of LDH became higher with increasing agarose content in the composite. The LDH moiety in the mixture showed low or even negative effect, which again confirmed that a homogeneous quasi-colloidal dispersion of LDH nanoparticles in the composite promoted chromate removal. Interestingly, increasing the amount of agarose in the composite enhanced LDH’s chromate scavenging capacity. As the amount of LDH in the composites was similar regardless of agarose concentration, this result could be explained by modified environment around LDH nanoparticles inside the hydrogel. Polysaccharide chains having δ^−^ sites can be weakly bound to the positive surface of LDH and thus, LDH nanoparticles could be sufficiently separated at high amount of the agarose moiety. Thus, it could be expected that increasing the agarose concentration enhanced the colloidal stability of LDH nanoparticles inside of the hydrogel. When the strongly negative chromate ions approached LDH, polysaccharides were readily replaced by them.

LDHs are known to have a high anion capacity, utilizing their positive surface. We verified that the interlayer carbonate can be partially exchanged by chromate (Figure S2). Thus, we could suggest that the chromate removal in current samples occurred by partial interlayer exchange as well as surface adsorption. Supposing that Mg_2_Al(OH)_6_(CO_3_)^2−^_0.5_-LDH with 20 nm size adsorbs anions on their surface only, an anion capacity of ~100 μmol(-)/g could be calculated. If that LDH particle uses interlayer space through an anion exchange reaction, the theoretical anion capacity could be as high as 4400 μmol(-)/g. The maximum chromate adsorption by the LDH itself (Figure 6) was larger than 1000 μmol(-)/g for all three composites (considering that chromate had two sites of negative charges, the values in Figure 6 was doubled.). Even the LDH moiety in C-2 showed a chromate adsorption of 2400 μmol(-)/g. This indicated that the LDH nanoparticles in the composite could adsorb chromate ions by multi-layer surface adsorption, partially utilizing their interlayer space. Thus, we fitted isotherm curves with Figure 5 to the well-established multi-layer adsorption Freundlich model using the equation below:

[*Q_e_ = K_F_·C_e_*^1/*n*^] (1)

where, *Q_e_*: adsorbed amount at equilibrium in mg/g unit, *C_e_*: chromate concentration at equilibrium in mg/L unit K*_F_*: the adsorption coefficient (*K_F_* = *K_adsorption_*/*K_desorption_*), *n*: a correlation coefficient indicating adsorption behavior.

The fitting result, displayed in Table 2, showed that the chromate removal of C samples was well-fitted to the Freundlich model, exhibiting a high coefficient of determination *R*^2^*_F_*. The correlation coefficient *n* is known to be related to the binding mode of adsorbate; adsorption is cooperative when *n* < 1 and anti-cooperative with *n* > 1. The coefficient *n* of the C samples were lower than 1, suggesting that the composite displayed cooperative multi-layer chromate adsorption tendencies. It was interesting that the *n* values of the composites decreased with increasing agarose content. As expected from Figure 6, increasing the agarose content could enhance the colloidal stability of the LDH, resulting in facilitated adsorption of chromate ions. The adsorption coefficient K*_F_* (= K*_adsorption_*/K*_desorption_*) revealed effective chromate removal of the C samples compared with corresponding M or A samples, exhibiting higher values than others.

**Table 2:** Freundlich adsorption model fitting results for agarose only hydrogels (A), LDH-agarose composite hydrogels (C) and LDH-agarose mixture hydrogels (M) samples.

Samples	*n*	*K_F_* (dm^3^g^−1^)	*R*^2^*_F_*
A-0.5	1.10	0.633	0.856
C-0.5	0.91	0.843	0.987
M-0.5	1.03	0.725	0.937
A-1	0.96	0.758	0.959
C-1	0.85	0.797	0.981
M-1	0.94	0.537	0.827
A-2	0.90	0.785	0.976
C-2	0.80	0.854	0.992
M-2	0.93	0.559	0.854

## 3. Experimental Section

### 3.1. Chemicals

Agarose (~120 kDa) was purchased from Bio Basic Canada Inc. (Toronto, ON, Canada). Magnesium nitrate hexahydrate (Mg(NO_3_)_2_·6H_2_O), aluminum nitrate nonahydrate (Al(NO_3_)_3_·9H_2_O), sodium bicarbonate (NaHCO_3_), and potassium chromate (K_2_CrO_4_) were purchased from Sigma-Aldrich Co. LLC. (St. Louis, MO, USA). Ammonium hydroxide (NH_4_OH) and hydrochloric acid (HCl) were purchased from Duksan Pure Chemicals Company (Ansan, Korea).

### 3.2. Preparation of Agarose Hydrogel (A), LDH-Agarose Composites (C), and LDH-Agarose Mixture (M)

Agarose hydrogels of different concentration were prepared with a 0.04 M carbonate buffer having pH of approximately 7.4. A designated amount of agarose powder was dispersed in 20 mL of prepared carbonate buffer, solubilized at 120 °C and cooled down in a cuboidal mold (4 × 3 × 1 cm^3^) to obtain 0.5, 1 and 2 wt/v% agarose hydrogels. Each hydrogel was named A-0.5, A-1, and A-2, corresponding to the 0.5, 1 and 2 wt/v% agarose concentrations, respectively.

In order to prepare LDH-agarose composites, each agarose hydrogel was located in the center of an electrophoretic kit dividing cationic (0.16 M Mg(NO_3_)_2_·6H_2_O and 0.08 M Al(NO_3_)_3_·9H_2_O) and anionic precursor (0.08 M NaHCO_3_ 250 mL and NH_4_OH 1 mL) solutions at the anode and cathode compartment, respectively. After applying a 25 V potential for 30 min, the agarose hydrogel containing LDH nanoparticles was washed by deionized water to remove surface attached ions. Each composite was named C-0.5, C-1, and C-2 for those starting from the 0.5, 1, and 2 wt/v% agarose hydrogels, respectively.

For comparison, LDH-agarose mixtures were prepared by simply adding LDH powder to hot agarose solution. LDH nanoparticles were prepared by a conventional co-precipitation route; Mg(NO_3_)_2_·6H_2_O (0.006 mol) and Al(NO_3_)_3_·9H_2_O (0.003 mol) were dissolved in deionized water (100 mL) and titrated with anionic solution (0.018 mol NaOH and 0.006 mol NaHCO_3_ in 250 mL) until pH ~9.5 with vigorously stirring and aged for 24 h at room temperature. The suspension was centrifuged, thoroughly washed with deionized water, and lyophilized. In order to prepare mixture samples, equivalent amount of LDH powder compared with C samples was added to hot agarose solution (0.5, 1, and 2 wt/v%) and stirred for 30 min, cooled down in cuboidal mold to form LDH containing hydrogel mixture, and then washed by deionized water. Each mixture was named M-0.5, M-1, and M-2, respectively.

### 3.3. Identification of LDH in Composites through Reconstruction

In order to identify the formation of LDH, we carried out a step-by-step calcination-rehydration process, also known as reconstruction. First, dehydrated hydrogels were calcined in a muffle furnace at 600 °C for 4 h to decompose the organic moiety of agarose and to transform LDH to MMO. The obtained MMO powder (~0.05 g), was dispersed in 50 mL a sodium bicarbonate solution (0.02 M) and reacted for 24 h under vigorous stirring. Finally, precipitates were collected by centrifugation, washed with deionized water and lyophilized.

The crystalline phases of the calcined composite (MMO) and reconstructed LDHs were identified by X-ray diffraction (XRD; D2 phaser, Bruker, Billerica, MA, USA) with Ni-filtered Cu-Kα radiation (λ = 1.5406 Å).

### 3.4. Characterization

Photographs of agarose and composite hydrogels, showing their overall feature, were taken by SONY NEX 5N in close-up mode. The LDHs’ particle size and distribution in the agarose and composite were observed using a scanning electron microscopic (SEM; FEI QUANTA 250 FEG, FEI company, Hillsboro, OR, USA) at 10 kV acceleration. For SEM analyses, each sample was dehydrated to a film and the surface of specimen was coated with Pt/Pd by sputtering for 30 s. The surface morphology of each material was evaluated using atomic force microscopy (AFM; NX10, Park systems, Suwon, Korea) in non-contact mode. For AFM measurement, each sample was slightly melted at 120 °C and cooled down on a flat silicon wafer.

In order to evaluate the dependence of the amount of LDH in the composite on agarose concentration, the mass of the solid part after calcination and reconstruction was recorded. After calcining composites (C-0.5, C-1, and C-2) at 600 °C, the mass of the residual powder was measured. As it is known that LDH (formula weight = 207.64 g/mol) transformed to MMO (formula weight = 131.59 g/mol) and again recovers LDHs during the reconstruction process [23], we applied a formula weight ratio to the mass of the MMO to estimate the mass of LDH in the composite.

### 3.5. Evaluation of Chromate Removal Efficacy of A, C, and M with Respect to Time and Concentration of Chromate

In the time-dependent chromate adsorption experiments, prepared hydrogels (A, C, and M) were soaked to chromate solution (400 ppm, 20 mL, pH 7.5). At the time point 0, 0.5, 1, 2, 4, 24, and 48 h chromate concentration in supernatant was quantified with a UV-VIS spectrophotometer (Shimadzu UV-1800, Shimadzu corporation, Kyoto, Japan) at λ_max_ = 372 nm. In order to obtain chromate removal isotherm, chromate stock solutions of 50 (pH ~6.94), 100 (pH ~7.04), 200 (pH ~7.35), and 400 ppm (pH ~7.51) was utilized. Each hydrogel sample was cut to 1 × 3 × 1 cm^3^ dimensions and soaked in the chromate solution (20 mL) for 24 h under gentle shaking. Equilibrium chromate concentration and adsorbed amount was evaluated with a UV-VIS spectrophotometer (Shimadzu UV-1800) at λ_max_ = 372 nm. For isotherm fitting analyses, a well-known adsorption model, the Freundlich equation [18], was applied (Equation (1) in Result and Discussion Section).

## 4. Conclusions

Composites were prepared containing quasi-colloidal LDH suspensions inside an agarose hydrogel matrix through electrophoretic preparation. It was revealed that LDH nanoparticles inside the hydrogel were homogeneously distributed with proper inter-particle distance, while the LDH-agarose mixture exhibited agglomeration of the LDH nanoparticles. Quantitative analyses showed that the starting agarose concentrations in the hydrogel did not significantly affect the amount of LDHs in the composites. Nevertheless, chromate removal efficiency of the composites was strongly affected by agarose content. Considering the adsorption of chromate by the LDH moiety in the composite or mixture, we could suggest that a higher agarose content facilitated stability of the quasi-colloidal state of LDH nanoparticles in the composite, resulting in enhanced chromate scavenging. From Freundlich adsorption fitting, composites were determined to have beneficial cooperative adsorption behavior with a high adsorption coefficient compared with agarose and mixture hydrogels.

## Supplementary Materials

They are available online at http://www.mdpi.com/2079-4991/6/2/25/s1.
