# Supplementary material for: Composites of Quasi-Colloidal Layered Double Hydroxide Nanoparticles and Agarose Hydrogels for Chromate Removal
Source: Nanomaterials (Basel). 2016 Jan 26;6(2):25. doi: 10.3390/nano6020025 (PMC5302482; doi:10.3390/nano6020025)
Supplement: Supplementary file 1 [file nanomaterials-06-00025-s001.pdf]

# Supplementary Materials

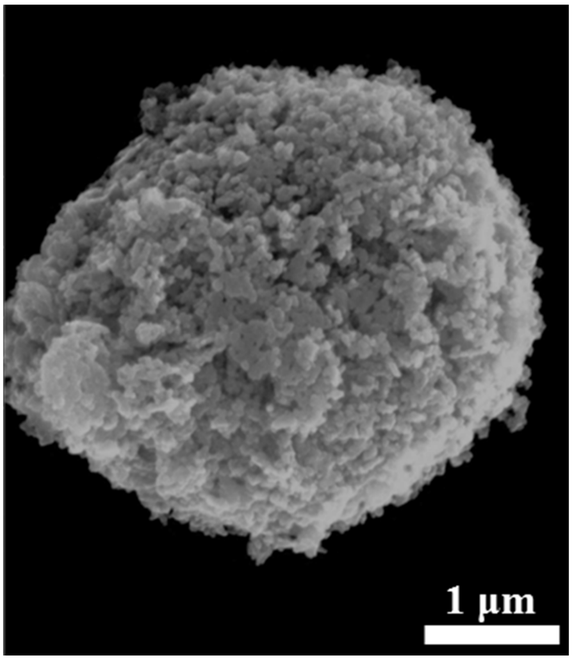


**Figure S1.** Scanning electron microscopic (SEM) image of Layered double hydroxide (LDH) nanopar ticles in powder form.

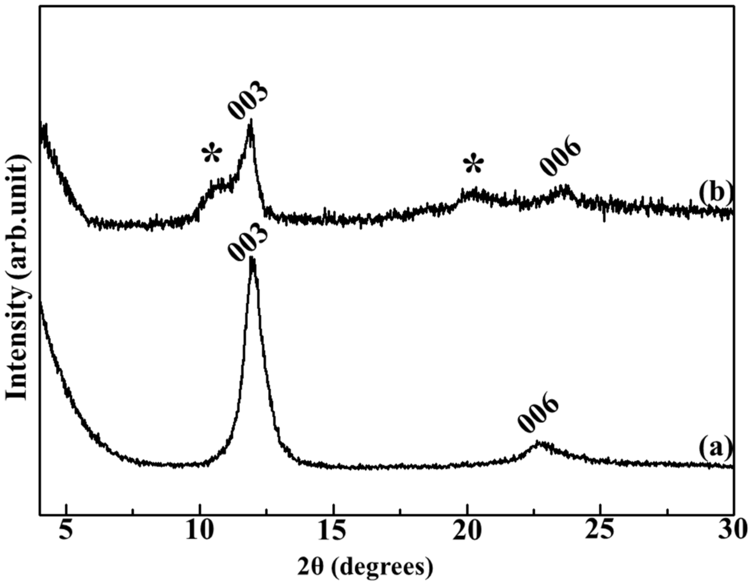


**Figure S2.** X-ray diffraction pattern of MgAl-$CO_3$-LDH (**a**) before and (**b**) after treated in chromate solution (1200 ppm). Asterisks (*) stand for the peak from LDH partially intercalated by chromate.

© 2016 by the authors; licensee MDPI, Basel, Switzerland. This article is an open access article distributed under the terms and conditions of the Creative Commons by Attribution (CC-BY) license (http://creativecommons.org/licenses/by/4.0/).
